# A Very Stable High Throughput Taylor Cone-jet in Electrohydrodynamics

**DOI:** 10.1038/srep38509

**Published:** 2016-12-05

**Authors:** M. R. Morad, A. Rajabi, M. Razavi, S. R. Pejman Sereshkeh

**Affiliations:** 1 Sharif University of Technology, Department of aerospace engineering, Tehran, Iran

## Abstract

A stable capillary liquid jet formed by an electric field is an important physical phenomenon for formation of controllable small droplets, power generation and chemical reactions, printing and patterning, and chemical-biological investigations. In electrohydrodynamics, the well-known Taylor cone-jet has a stability margin within a certain range of the liquid flow rate (*Q*) and the applied voltage (*V*). Here, we introduce a simple mechanism to greatly extend the Taylor cone-jet stability margin and produce a very high throughput. For an ethanol cone-jet emitting from a simple nozzle, the stability margin is obtained within 1 kV for low flow rates, decaying with flow rate up to 2 ml/h. By installing a hemispherical cap above the nozzle, we demonstrate that the stability margin could increase to 5 kV for low flow rates, decaying to zero for a maximum flow rate of 65 ml/h. The governing borders of stability margins are discussed and obtained for three other liquids: methanol, 1-propanol and 1-butanol. For a gravity-directed nozzle, the produced cone-jet is more stable against perturbations and the axis of the spray remains in the same direction through the whole stability margin, unlike the cone-jet of conventional simple nozzles.

Rayleigh proposed a theory on a spherical liquid droplet subjected to an electric field for the first time showing that the spherical shape could not stay stable beyond a threshold of electric potential difference^1^. Later, Taylor defined a cone-jet mode for a range of applied voltage corresponding to a stable conical liquid meniscus at the nozzle exit with a tiny jet at the end^2^. The physical mechanism is studied^3^,^4^ with its wide application in physics of small droplets^5^,^6^,^7^,^8^,^9^,^10^,^11^,^12^,^13^,^14^. High resolution printing and fabrication are two examples of electrohydrodynamically induced jets applications^10^. Insulating or conducting polymers^10^ and Graphene as a 2D highly conductive material can be printed with high resolution on planar or highly curved surfaces^15^ using this technique. Fabrication of various devices like organic light-emitting diodes^16^ and biosensors^17^ are further examples of electrohydrodynamic printing, which require production of fine uniform drops with great control.

For every liquid with given properties, there is a stability island in terms of flow rates and potential differences in which the cone-jet stay stable^18^,^19^. The cone-jet only stabilizes in a limited range of flow rates reducing the benefit of electrospray in different applications^18^,^20^,^21^,^22^. Stability of the cone-jet is crucial for combustion of liquid fuels in small scales^8^,^20^,^23^, while in meso/micro scales it is important to produce very fine droplets but sufficiently large mass flow rates. The maximum and minimum flow rates of the stability island depend on liquid conductivity^24^. The range of voltage which may lead to a stable cone-jet structure depends on nozzle geometry, electrodes configuration, flow rate, and liquid properties especially conductivity and surface tension.

The stable cone-jet mode is confined between two boundaries at each flow rate: the upper voltage boundary between the cone-jet and multi-jet modes, and the lower voltage boundary between the unstable spindle and the stable cone-jet modes. The spacing between high and low voltage boundaries reduces to zero as flow rate increases and a stable cone-jet can only be achieved if the flow rate is sufficiently low. The multi-jet mode produces finer droplets but with a broader size distribution compared to the cone-jet^25^.

For ethanol and other suspensions, the maximum stable flow rate was increased by modifying the electric field using a third electrode called the collector electrode^26^. Controlling the electric field near the nozzle tip could significantly increase the voltage range of cone-jet stability and alter the angle of the spray plume as well^27^. A number of efforts on changing the specifications of the nozzle for having more control on the cone-jet mode is reported, utilizing a ballpoint pen^28^, carbon fiber^29^ and externally wetted emitters^30^.

The mechanism that determines minimum and maximum flow rates of the cone-jet mode is ambiguous^31^. We discuss the governing physical mechanism and introduce a novel yet simple emitter in order to enhance stability of the cone-jet mode for a wide range of flow rates and applied voltages. This extension in flow rates is provided by adding a small part, called the “extender cap” hereafter, to the conventional simple nozzle. The stability margins are determined experimentally for four common alcohols, namely methanol, ethanol, 1-propanol and 1-butanol, comparing the new emitter performance with a simple nozzle.

## The Extender Cap Cone-Jet

**Figure 1:**
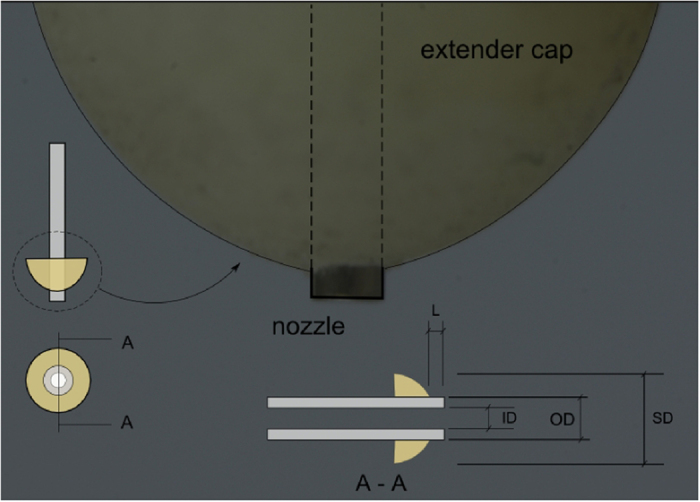
Geometry of the extender cap nozzle. The hemispherical extender cap is made from ABS and is installed near the tip of the steel nozzle with a small recess (*L*). The dimensions are listed in Table 1.

**Figure 2:**
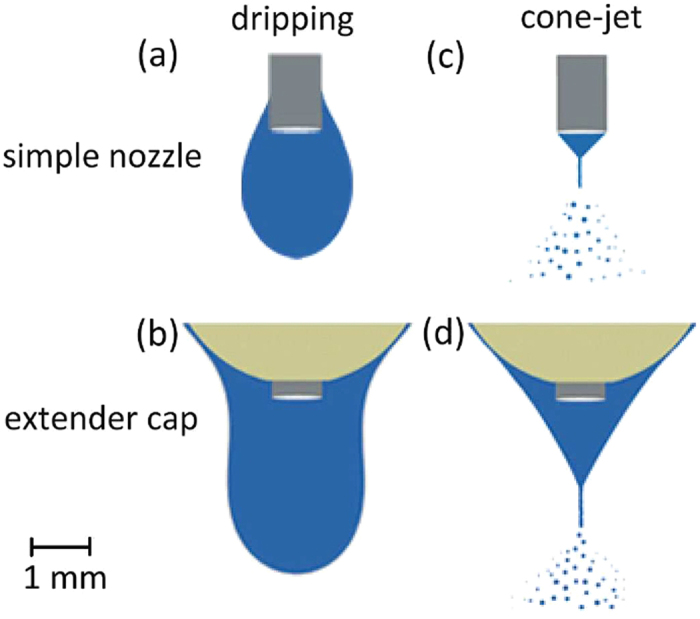
The liquid rises the wettable nozzle outer wall in the dripping mode (a). After installation of the extender cap, the recess was small enough for rising droplet to reach the extender cap and adhere to its bottom surface (**b**). When the electric field is intensified the cone is formed with its base connected to the extender cap (**d**), instead of the nozzle tip itself (**c**).

A hemispherical extender was designed to be installed near the tip of a simple nozzle. The extender was produced from nonconducting ABS (Acrylonitrile butadiene styrene) with a central passage through which the nozzle could be installed. The geometry is shown in Fig. 1 and the related dimensions are listed in Table 1. When no electric field existed, the liquid climbed up the wettable simple nozzle due to capillary force and eventually pinched off when it is dominated by the gravity force^32^. After installation of the extender cap, the liquid interacted with the outer wall of the cap and, consequently, a larger droplet formed before the pinch off (see Fig. 2). The effect of solid surfaces can be taken into account for a stable pendant drop where there are liquid-solid-vapor interfaces^33^,^34^,^35^. A vertical force balance equation can be written at ceiling surfaces, where the mean liquid pressure exists over the liquid anchoring area (both for liquid and solid ceiling). The upward component of the surface tension acts along the solid-liquid-vapor contact line, and the vertical pressure gradient along the vertical axis of symmetry must be satisfied as *dp*/*dz* = −*ρg*. After integration, the difference in capillary pressure between the top and the bottom of the drop is due to the difference in gravitational potential energy. So, the drop will form with a fixed contact angle. Mentioned balance equations can explain why the extender cap can support a larger liquid volume that covers the metallic nozzle tip.

**Figure 3:**
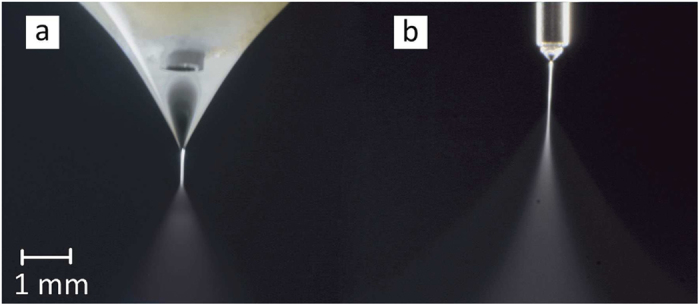
The cone-jet mode of ethanol electrospraying from an extender cap (**a**) at *Q* = 5 ml/h and *V* = 7.3 kV and a simple nozzle (**b**) at *Q* = 2 ml/h and *V* = 4.3 kV. The steel cylindrical nozzles are identical.

When the electric field presents, there is a minimum voltage for which the curvature of the drop surface converts to a cone called the Taylor cone. The Taylor cone-jet when emitted from a conventional simple nozzle and from a nozzle extended by a spherical cap are illustrated in Fig. 3, for comparison. As previously mentioned, there is an interaction between the liquid and the extender cap, which is maintained after the cone-jet is formed. The extender cap results in a much broader stability of the cone-jet mode for much higher flow rates. Also, it is observed that the cone-jet formed with the extender cap is much more stable compared to the simple nozzle when both of them operate at the same flow rate.

## The Stability Island and the Jet Diameter

**Figure 4:**
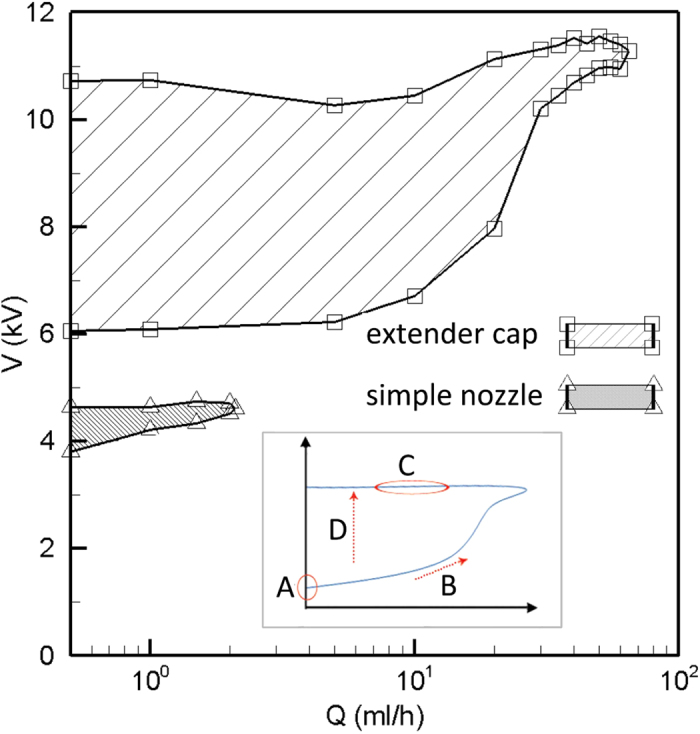
The shaded regions show stability islands corresponding to the two nozzles. The inset shows a typical stability island with the corresponding zones.

Here, the characteristics of ethanol cone-jet using both simple and extender cap nozzles are presented in detail. Results of other liquids are provided for comparison, too. The stability island of the ethanol cone-jet mode is depicted in Fig. 4 in which the flow rate (*Q*) is plotted versus the applied voltage (*V*). For the same nozzle inner and outer diameters and fixed electrode separation distance, the plots reveal that the extender cap enlarges the maximum flow rate of the cone-jet mode to about 65 mL/hr, compared to the maximum flow rate of 2 mL/hr for the nozzle alone.

**Figure 5:**
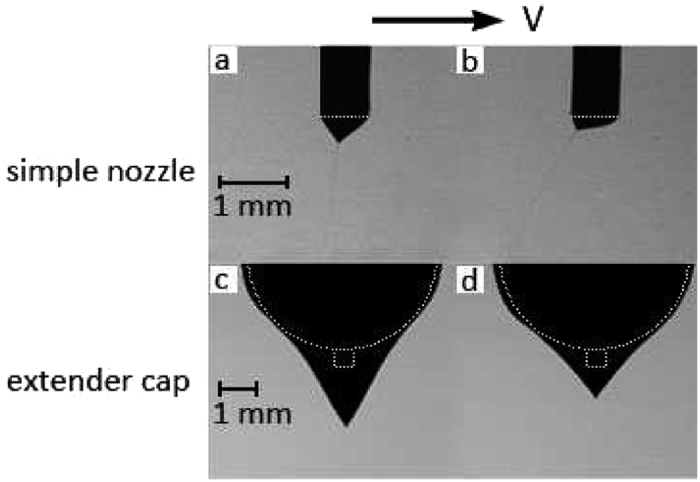
Comparison of the jet direction for ethanol at *Q* = 1.5 ml/h. The voltage varies between 4 (**a**) to 4.2 kV (**b**) for the simple nozzle and between 6.4 (**c**) to 7.5 kV (**d**) for the extender cap nozzle. The jet does not deviate from the nozzle axis with increasing voltage in the extender cap nozzle unlike the simple nozzle. The solid boundaries are depicted with white dashed lines. Both scales indicates 1 mm length, and the simple nozzle segments are double sized for clarity.

Each flow rate is associated with a minimum voltage where the cone-jet mode is stabilized, and a maximum voltage beyond which the cone-jet is destabilized. Depending on the surrounding gas as well as the electrode configuration, the maximum voltage may be limited either by corona discharge or a transition to multi-jet mode. The cone-jet is more stable at low flow rates, which means that a wider range of voltage differences between the nozzle and the ground electrode could produce a stable cone jet in lower flow rates. In the conventional simple nozzle configuration, the cone-jet voltage band is about 1 kV for low flow rates tending to narrow to zero at the maximum flow rate. At 0.5 mL/hr a cone-jet structure could be observed between 3.7 kV to 4.6 kV for the conventional configuration. For the extender cap and at 0.5 mL/hr, the cone-jet appears between 6 and 10.6 kV. For all flow rates, the voltage band that could form a Taylor cone structure is much broader for the extender cap nozzle. At low flow rates, the difference between upper and lower limit is about 5 kV, with a *V*_*max*_/*V*_*min*_ ratio of about 1.8, which is reported to be a quantity of order one for conventional nozzle configuration^31^. The steadiness of the cone-jet structure is also important in various applications regarding producing uniform droplet sizes. It was observed that the extender cap nozzle could sustain a more stable cone-jet structure compared to the simple nozzle, which is kind of sensitive to disturbances. Particularly, using the simple nozzle the jet deviates from the injection axis at high voltages. However, the extender cap nozzle keeps the jet in the injection axis up to the maximum voltage boarder (see Fig. 5).

The extender cap nozzle is observed to be effective in stabilizing the cone-jet structure with other common alcohols as well. Methanol, 1-propanol, and 1-butanol were investigated in addition to ethanol and results of stability margins could be compared in Table 2. The maximum flow rate at which a stable cone-jet can form and the voltage band of the stability island at *Q* = 1 mL/hr is reported in the table. Noteworthy, no upper limit was observed for 1-propanol and 1-butanol in terms of flow rate. The present observations showed that the cone-jet converts into a simple jet at higher flow rates for 1-propanol and 1-butanol when an extender cap nozzle is used. The reported range of flow rate for all liquids belongs to the region where the minimum voltage boarder of the stability island has a positive slope, which will be justified in the discussion section. Significant increase in the cone-jet stability limits were observed in terms of flow rate and voltage band for all four tested liquids.

**Figure 6:**
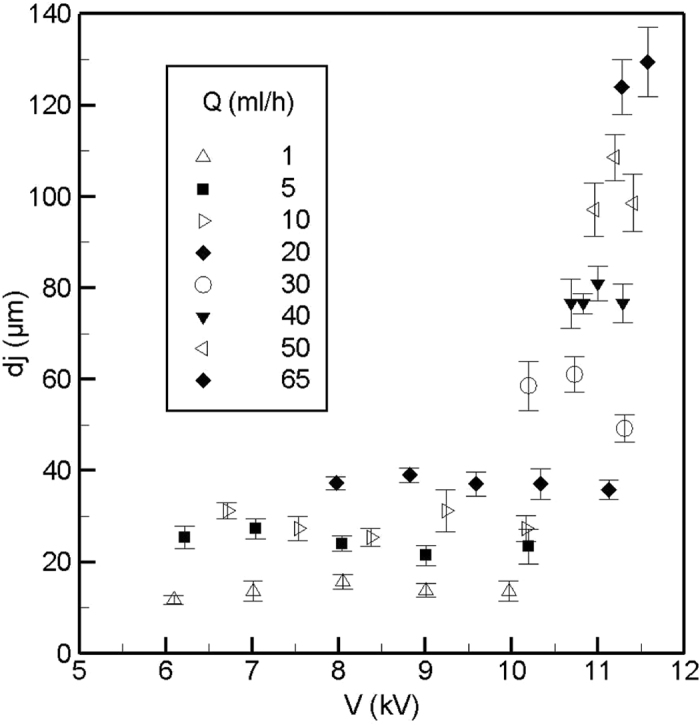
The diameter of the ethanol jet which emerges from the Taylor cone tip increases with the flow rate but is almost independent of the voltage. At large flow rates, the voltage band of the stability island is narrow and the number of data is limited.

It is also of interest to investigate the jet diameter (*d*_*j*_) of the con-jet from the extender cap and compare it with the one of the simple nozzle. The jet diameter of a simple nozzle is controlled with the flow rate rather than the voltage as reported in the literature^36^. Figure 6 shows the jet diameter for different flow rates and applied voltages for the extender cap nozzle. The jet diameter increases with the flow rate but varies slightly at different voltages as can be seen in the figure. The jet diameter is almost independent of the applied voltage especially for flow rates less than 30–40 mL/hr. For higher flow rates, the voltage band is quite small and it was observed that the Taylor cone shows short amplitude oscillations in the direction of the nozzle axis. The oscillations do not change the cone jet structure but alters the jet diameter. The jet diameters for the simple and extender cap nozzles are not significantly different at the same flow rates as was observed in the present study.

**Figure 7:**
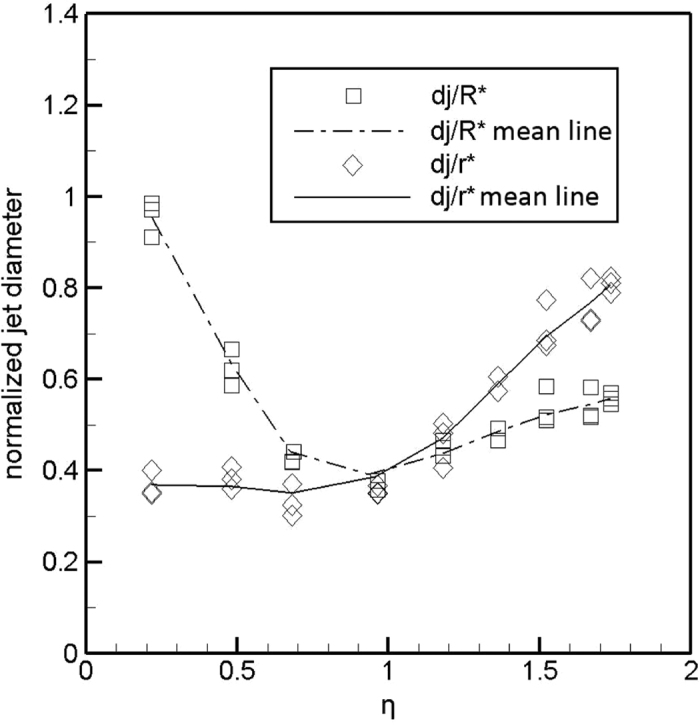
The ratio (*d*_*j*_/r^*^) is almost independent of *η* for *η* < 1, meaning that the r^*^ could be an appropriate scale for the jet diameter at low flow rates. The ratio (*d*_*j*_/R^*^) decreases with *η* for *η* < 1 but varies slightly when *η* > 1. The proper scale for the jet diameter at high flow rates is R^*^. By definition r^*^ = R^*^ at *η* = 1. The jet diameter is measured for three different voltages at each *η* and the lines represent the mean values trend.

The jet diameter can be scaled with R^*^ = (*ρQ*^2^/*γ*)^1/3^ considering a balance between inertia and surface tension forces^36^, in which R^*^ is a characteristic jet radius, *ρ* is the liquid density, *Q* is the volume flow rate and *γ* is the surface tension. Non-dimensionalized jet diameter (*d*_*j*_/R^*^) is obtained versus *η* as plotted in Fig. 7, where *η* is defined as (*ρKQ*/*γε*)^1/2^ and represents the square root of a non-dimensionalized flow rate. *K* is the liquid conductivity and *ε* is its electrical permittivity. (*d*_*j*_/R^*^) varies slightly when flow rate is *η* > 1, as can be seen in Fig. 7. At low flow rates, as well as, when flow rates decrease towards zero, *d*_*j*_/R^*^ rises extensively. The jet diameter can scaled with two different charactristic radii, R* and r^*^ = (*Qε*/*K*)^1/3^ which is shown in the same figure. This length scale originates from a balance between the electrical normal stress and the surface tension at the tip of the cone and is related to the charge relaxation phenomena. It can be seen that *d*_*j*_/r^*^ is nearly independent of the flow rate for *η* < 1, as also previously discussed for simple nozzles^36^. Thus, for the new nozzle configuration with the extender cap, it may be concluded that at low flow rates the jet is formed as a result of the charge relaxation phenomena, while at high flow rates it is formed as a result of the inertia disturbing balance between surface tension and electrical forces. This behavior is akin to that of the cone-jet structure of conventional simple nozzle configuration. Therefore, there is a high probable that the same balance of forces are responsible for the cone-jet stabilization in the conventional simple nozzle and the extender cap configurations.

## Discussion

In this section, we will discuss the characteristics of the cone-jet stability island for a conventional simple nozzle configuration and the effect of the extender cap in more details. This will be done by examining the balance of forces acting on the cone-jet structure and contemplating our observations of the behavior of this structure at its stability boarders, as well as, examine discussions made by others. The Navier-Stokes equation is written in a one-dimensional form for a cone-jet as follows (a diagram of forces is shown in Fig. 1 of Hartman’s publication cited here)^37^.


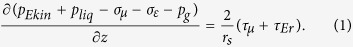


Here, *p*_*Ekin*_ is the velocity pressure, *p*_*liq*_ is the liquid pressure, *σ*_*μ*_ and *τ*_*μ*_ are the normal and tangential viscous stresses respectively, *σ*_*ε*_ is the polarization stress, *p*_*g*_ is the hydrostatic pressure, and *τ*_*Er*_ is the tangential electric stress. The liquid pressure have the following terms.


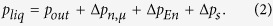


The surrounding air pressure is *p*_*out*_, the stress on the liquid-gas interface due to viscous effects is Δ*p*_*n*,*μ*_, the electrical normal stress on the interface is Δ*p*_*En*_, and the surface tension stress is Δ*p*_*s*_. These two equations along with the charge balance on the liquid surface have been shown to be able to predict the characteristics of the cone-jet phenomenon well. This model takes into account all forces, but some of them are dominant in the margins of the stability island. The cone-jet shape is mainly a result of a balance between the velocity pressure, the surface tension stress, the electrical normal and tangential stresses^38^,^37^. In the following the underlying physics that determines the minimum and maximum voltage boarders of the cone-jet stability island is argued, considering disappearance of the cone-jet structure at very high flow rates.

For the onset of the cone-jet formation associated with minimum flow rates (see A in the inset of Fig. 4), it is known that the surface of a pendant droplet will deform into a cone when the normal electrical stress on the liquid surface Δ*p*_*En*_ becomes large enough to counter balance the surface tension stress Δ*p*_*s*_^2^. Using the mentioned force balance, the required voltage for the onset of the cone formation will be accurately predicted based on the liquid surface tension and electrodes geometry^39^. Consider a pendant drop from a simple nozzle with its axis positioned vertically downward towards the gravity vector, while the surface of the pendant drop is a concave curvature. Upon applying a uniform downward electric field, the droplet surface starts to deform to a cone when the applied voltage approaches the minimum value required for the cone-jet mode. After the formation of the cone, a reduction of the voltage back below the minimum voltage reduces the electric field intensity on the liquid surface, however the conical form will be maintained as the voltage decreases below the minimum boarder. This hysteresis in cone-jet formation is observed in the present experiments and is also reported by other researchers^25^,^40^. It is interesting to note that the deformation of the liquid surface from a concave curvature into a conical shape increases the average contribution of the normal component in the direction of the main electric field over the droplet surface. For any cone semi-angle (*θ*) greater than 35 degrees, a simplified check shows that the average normal electrical pressure acting on the cone surface is greater than that of a semi spherical droplet by a factor of 3sin^2^ *θ*, neglecting electric field variations due to the presence of the liquid meniscus. The observed cone semi-angle at the minimum voltage boarder was generally greater than 35 degrees. Therefore, the cone-jet mode hysteresis may be explained as being a consequence of the decomposition of the electric stress into normal and tangential components as a result of the conical shape formation.

The onset voltage of the cone-jet formation increases with volume flow rate (along B in the inset of Fig. 4). Remarkably, the velocity pressure *p*_*Ekin*_ is the main significant term that varies with the flow rate in the 1-D force balance, equation 1, and it could be responsible for the mentioned behavior of the minimum voltage boarder. Note that the viscous terms *σ*_*μ*_ and *τ*_*μ*_ are not dominant. The force needed to accelerate the fluid toward the cone tip *F*_*k*_, is the difference between the velocity pressure (specific kinetic energy) cross the flow area A from the cone base to its tip.


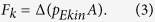


**Figure 8:**
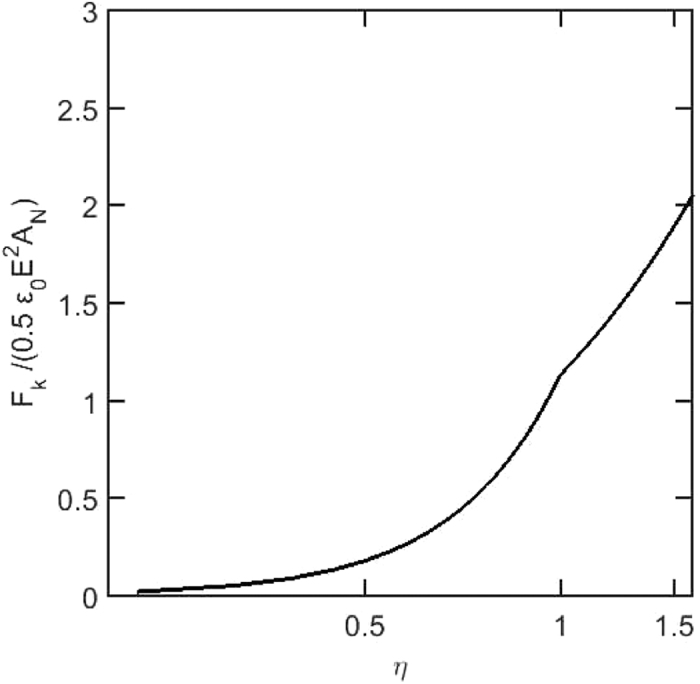
The force needed to accelerate the liquid toward the cone tip, increasing with the nondimensional flow rate *η*.

*F*_*k*_ increases with the flow rate and its correlation could be simply obtained if the velocity profile at the nozzle exit and in the jet region are assumed to be uniform and the jet area is calculated based on the measured jet radius. Thus, zero-dimensional analysis yields the force as:


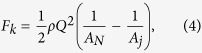


in which *ρ* is fluid density, *A*_*N*_ is the nozzle exit area, and *A*_*j*_ is the jet cross sectional area. As mentioned previously, when *η* < 1, *A*_*j*_ increases with *Q*^(2/3)^ (or *r*_*j*_ increases with *Q*^(1/3)^). Otherwise, *A*_*j*_ increases with *Q*^(4/3)^ (or *r*_*j*_ increases with *Q*^(2/3)^)^36^. The force needed to accelerate liquid in the present experiments is calculated for a range of liquid flow rates and is depicted in Fig. 8. The force *F*_*k*_ is nondimensionalized with a characteristic electric force of 0.5*ε*_0_*E*^2^*A*_*N*_ and the flow rate is shown with *η* in the figure. The characteristic electric field calculated between the nozzle and the counter electrode with a voltage of *V* = 8 kV. The change in the slope of *F*_*k*_ at *η* ≈ 1 (*Q* ≈ 20) is due to the change in the mechanism of the jet formation. Above *η* ≈ 1 the inertia characteristic radius R^*^ becomes dominant over the charge relaxation characteristic radius r^*^. The jet emerges as a result of the inertia, breaking the equilibrium between the surface tension and the electric stresses at the cone tip^36^. The same discussion would be held for the cone-jet emitted from the extender cap since the characteristics and mechanism of both are similar. Most noteworthy, is the change in behavior of the slope of the minimum voltage boarder for the stability island of the cone-jet from the extender cap (Fig. 4) also occurs at *Q* ≈ 20 (*η* ≈ 1) like the slope of the *F*_*k*_ from the present analysis (Fig. 8).

The tangential electric stress *τ*_*Er*_ on the liquid cone causes the acceleration of the liquid from the nozzle toward the cone tip^4^,^41^. Results from the cone-jet physical modeling have shown that an increase in momentum of the liquid is mainly due to the tangential electric stress acting on the cone surface among other forces^37^,^42^. As argued above, increasing the liquid flow rate in the cone-jet mode results in an increasing need for tangential electric stress to accelerate the flow. Consequently, the minimum voltage required to stabilize the cone-jet structure increases as the flow rate increases.

Regarding the maximum voltage border of the cone-jet stability island (see C in the inset of Fig. 4), it is known that this boarder is associated with the appearance of multi-jet mode or the corona discharge^31^. Corona discharge is an electrical discharge through the surrounding gas and is less dependent on the liquid flow rate. A multi-jet mode may appear before the corona discharge. Typically, when the voltage increases the space charges skews the axisymmetric cone and a single jet emerges at the rim of the nozzle. This is an unstable situation and eventually multi-jet mode appears when the meniscus is nearly flattened to the nozzle face and two or more micro cones appear around the rim symmetrically, each of them emitting a jet^43^.

The transition to the multi-jet mode is a result of the new balance of the involved forces. The observed maximum voltage boarder of the cone-jet stability island in the present study was associated with a transition to the multi-jet mode and was almost independent of *Q* (see Fig. 4), which is also reported by other scholars^26^,^44^. This independence indicates that the mechanism which is responsible for the multi-jet formation is not governed by the velocity pressure. The balance is otherwise between the electrical normal stress and surface tension stress. This argument is supported by the fact that the cone-jet to multi-jet transition occurs by increasing the electrical field intensity at the nozzle exit region, even at a constant surface charge density. This is examined in a numerical simulation^45^, where only an increase in the voltage of the ring electrode showed to be enough for the transition to multi-jet mode. In that study all nondimensional parameters representing the relative magnitude of inertia versus viscous, gravity, surface tension, polarization, and columbic forces were kept constant.

The behavior of the liquid meniscus when the voltage increases from the minimum value that onsets the cone-jet structure to the maximum boarder above which the multi-jet mode appears at fixed flow rate (along D in the inset of Fig. 4) may be explained in the following manner. Increasing the voltage above the minimum voltage at a particular flow rate results in reduction of the cone height with higher cone angles^40^. Since the jet diameter of a stable cone-jet structure is independent of electrostatic variables^36^,^46^, there is no need for tangential electric stress to accelerate the liquid more than the required value for the minimum voltage at the same flow rate. Therefore, the tangential stress on the interface is adjusted by increasing the cone angle. The overall electric field intensity and the contribution of the normal electric field component increase on the liquid surface as the voltage and the cone angle increase, until the maximum voltage is reached and the multi-jet mode appears. Since the normal electric stress balances the surface tension in the cone-jet mode^2^,^37^, the appearance of micro cone-jet structures on the interface in the multi-jet mode is likely a result of the high normal electrical stress which is more intensified around the nozzle rim and establishes a number of new local equilibrium near the rim.

In summary, an interaction between the electrical normal stress and the surface tension determines the onset voltage of the cone-jet mode for the minimum flow rate. Increasing the flow rate requires an increased shear stress to accelerate it which means an increased minimum voltage for the cone-jet stabilization. On the other hand, increasing the voltage at a constant flow rate allows the shorter cone with a larger apex angle to maintain the constant flow acceleration. Maximum voltage boarder is where the large electric normal stress breaks the existing balance with the surface tension locally at the rim periphery creating multiple micro cone-jets on the liquid meniscus. The maximum flow rate of the cone-jet stability island is the point where the minimum voltage required for the cone-jet stabilization reaches the prescribed maximum voltage. In fact, the maximum flow rate of a stable cone-jet is restricted by the maximum voltage boarder.

In regard to the cone-jet emitted from the extender cap, the large liquid volume suspended from the extender cap covers the nozzle tip that serves as the high potential electrode. In the extender cap configuration the electric field intensity on the liquid-air interface is substantially lower than that of the simple nozzle at the same voltage. Hence, a larger potential difference between the two electrodes is required to provide the equilibrium between the electric normal stress and the surface tension. Consequently, the cone-jet minimum voltage is larger for the extender cap nozzle (Fig. 4). The electric field reduction on the liquid interface also occurs at the maximum voltage boarder, compared to the conventional simple nozzle, but not much in the same way. It should be noted that, unlike the conventional simple nozzle, the liquid cone of the extender cap is not flattened to the nozzle level even at high voltages (see Fig. 5). This is due to the spherical shape of the extender cap as well as the nozzle recess with respect to the extender cap (see Fig. 1). In the extender cap configuration the electric field retains both normal and tangential components, even at high potential differences. The retained tangential component leads to more fluid acceleration while preventing the excessive growth of the normal component. Therefore, the normal electric field domination over surface tension and transition to the multi-jet mode is postponed upon using the extender cap and the voltage band broadens. It is noteworthy that using a conducting guard plate has been shown to broaden the voltage band by reducing the electric field intensity near the nozzle tip^27^, which is comparable to the present mechanism. The larger interval between the minimum and maximum voltages in the extender cap nozzles allows for a much larger maximum flow rate for the cone-jet mode.

In conclusion, the extender cap configuration makes it possible to extend the cone-jet stability island by formation of a large suspended liquid droplet covering the nozzle tip. This liquid volume modifies the electrical field and electric stresses on the liquid surface, enlarging the margin between the minimum voltage required to form a cone-jet and the maximum voltage that destabilizes it, leading to a broader flow rate margin.

## Methods

The liquid was supplied by a calibrated syringe pump while high voltages were applied between the nozzle and a plate. Applied voltages were measured using a high voltage probe and a digital multimeter with an accuracy of 0.1%. The liquid meniscus was visualized by a high speed CCD camera (1000 FPS, AOS technology) and a digital camera (D7100, Nikon) combined with a lens (Micro-Nikkor 105 mm f/2.8G from Nikon) and three automatic extension tubes (12, 20, 36 mm, Kenko). The set provided a maximum magnification of 1.65 with a spatial resolution of 2.3 *μm* for diameter measurements. Jet diameters reported in this paper are averaged values of four images with a mean standard deviation of 3 *μm*. A white LED was used as an illuminating light source for capturing images. The nozzle was a stainless steel one with an outer diameter of 0.7 mm and an inner diameter of 0.5 mm and the counter electrode (an aluminum plate of 100 × 100 × 2 mm) was fixed at 35 mm from the nozzle tip. Four liquids were used as working fluids. The physical properties of liquids is listed in Table 3. The basic existing nozzle for emitting the electrospray was a simple cylinder.

## Additional Information

**How to cite this article**: Morad, M. R. *et al*. A Very Stable High Throughput Taylor Cone-jet in Electrohydrodynamics. *Sci. Rep.* **6**, 38509; doi: 10.1038/srep38509 (2016).

**Publisher's note:** Springer Nature remains neutral with regard to jurisdictional claims in published maps and institutional affiliations.

## Figures and Tables

**Table 1:** Dimensions of the extender cap nozzle shown in Fig. 1.

Inner Dia. (ID) [mm]	Outer Dia. (OD) [mm]	Sphere Dia. (SD) [mm]	Recess (L) [mm]
0.4	0.7	6.2	0.2

**Table 2:** Maximum flow rates and voltages for the cone-jet margin for different liquids.

Liquid	*Q_max_* with the simple nozzle [mL/hr]	*Q_max_* with the extender cap [mL/hr]	Δ*V* at 1 mL/hr for the simple nozzle [kV]	Δ*V* at 1 mL/hr for the extender cap [kV]
Ethanol	2.4	65	0.5	4.9
Methanol	1.9	32	0.7	2.9
1-Propanol	2.8	70	0.6	5.5
1-Butanol	3.4	80	0.4	6.5

**Table 3:** Properties of the liquids.

Liquid	Conductivity [Sm^−1^]	Surface Tension [Nm^−1^]	Viscosity [mPas]	Density [kgm^−3^]
Ethanol	7.0*E*–6	0.024	1.2	789
Methanol	7.0*E*–6	0.021	0.59	795
1-Propanol	9.0*E*–6	0.024	1.94	803
1-Butanol	6.0*E*–6	0.023	2.54	800
